# Sepsis‐associated encephalopathy: Autophagy and miRNAs regulate microglial activation

**DOI:** 10.14814/phy2.15964

**Published:** 2024-03-05

**Authors:** Nannan Qin, Yanmei Miao, Leiyu Xie, Xinglong Ma, Peng Xie

**Affiliations:** ^1^ Department of Critical Care Medicine of the Third Affiliated Hospital (The First People's Hospital of Zunyi) Zunyi Medical University Zunyi China

**Keywords:** autophagy, microglia, microRNA, neuroinflammation, sepsis‐associated encephalopathy

## Abstract

Sepsis‐associated encephalopathy (SAE) describes diffuse or multifocal cerebral dysfunction caused by the systemic inflammatory response to sepsis. SAE is a common neurological complication in patients in the middle and late stages of sepsis in the intensive care unit. Microglia, resident macrophages of the central nervous system, phagocytose small numbers of neuronal cells and apoptotic cells, among other cells, to maintain the dynamic balance of the brain's internal environment. The neuroinflammatory response induced by activated microglia plays a central role in the pathogenesis of various central nervous system diseases. In this paper, we systematically describe the functions and phenotypes of microglia, summarize how microglia mediate neuroinflammation and contribute to the occurrence and development of SAE, and discuss recent progress in autophagy‐ and microRNA‐mediated regulation of microglial activation to provide a theoretical basis for the prevention and treatment of SAE and identify related therapeutic targets.

## 1 INTRODUCTION

Sepsis refers to life‐threatening organ dysfunction caused by dysregulation of the host response to infection by various pathogens and is one of the main causes of increased mortality in intensive care units (Hollenberg & Singer, 2021). Sepsis‐associated encephalopathy (SAE) describes diffuse or multifocal cerebral dysfunction of the brain caused by sepsis in the absence of clinical and laboratory evidence of direct infection, structural brain damage, or other types of encephalopathy (Gofton & Young, 2012; Ren et al., 2020). SAE is one of the main diseases that increases the mortality rate of patients in intensive care units, especially elderly patients; the mortality rate of SAE increases with severity and can even reach 70% (Catarina et al., 2021; Iwashyna et al., 2010). Therefore, early diagnosis and treatment of SAE are particularly important for reducing mortality rates.

Currently, the underlying molecular mechanisms of SAE are unclear, but they may be related to microglial hyperactivation, neuroinflammation, blood–brain barrier disruption, oxidative stress, neurotransmitter dysregulation, ischemic hypoxic injury, synaptic dysfunction, and abnormal blood flow regulation. Neuroinflammation induced by microglial hyperactivation is the core mechanism underlying the pathogenesis of SAE and is likely one of the main causes of sepsis‐associated brain dysfunction (Gao et al., 2022; Mazeraud et al., 2018; Moraes et al., 2021). Currently, there are no clear diagnostic criteria for SAE; moreover, while inflammation and SAE symptoms can be alleviated, there are no specific treatments for SAE. Therefore, the pathogenesis of SAE needs to be further explored, and effective treatment options need to be developed.

## 2 PHYSIOLOGICAL CHARACTERISTICS OF MICROGLIA

Microglia are glial cells that make up approximately 5%–12% of all cells in the human brain (Jin et al., 2019). Microglia originate from myeloid progenitor cells (primitive macrophages) in the embryonic yolk sac and are resident innate immune cells of the central nervous system (Ginhoux & Garel, 2018; Nayak et al., 2014; Subhramanyam et al., 2019). Like peripheral macrophages, microglia can rapidly and efficiently remove pathogens, dead cells, cellular debris, abnormal proteins and small numbers of neuronal cells through phagocytosis, playing an important role in maintaining the dynamic balance of the central nervous system and in disease processes, as well as in normal development of the brain (Borst et al., 2021; Gaudet & Fonken, 2018; Kierdorf & Prinz, 2017; Subhramanyam et al., 2019). During normal brain development, microglia contribute to brain development and homeostasis by interacting with various neuronal and nonneuronal cell types (Mehl et al., 2022). Microglia‐mediated synaptic pruning involves the maintenance of synaptic turnover, elimination of unwanted synapses, and establishment of neuronal circuits that have not previously been found (Andoh & Koyama, 2021; Paolicelli et al., 2011). Microglia are involved in regulating the maintenance and regeneration of myelin, the membrane that surrounds neuronal axons, and is necessary for axonal health and function in the central nervous system. Myelin sheaths are damaged with normal aging and in a variety of neurodegenerative diseases, such as multiple sclerosis and Alzheimer's disease (Berghoff et al., 2021; Kent & Miron, 2024; Lloyd & Miron, 2019; Yamanaka et al., 2023). Under physiological conditions, resting microglia (M0 microglia) have relatively long cytoplasmic protrusions and exhibit a branched morphology; they interact with surrounding neurons and other cell types, constantly monitor the central nervous system (CNS) and sense and respond to changes in the microenvironment, while also coordinating neuroinflammation through the secretion of important immune mediators (Jin et al., 2019).

## 3 MICROGLIAL ACTIVATION AND NEUROINFLAMMATION

### 3.1 Microglial activation

Microglia shift from a resting state (branched) to an activated state (amoeboid) in response to endogenous or exogenous stimuli such as lipopolysaccharide, cellular debris, or blood–brain barrier damage. Upon activation, the secretion pattern of microglia changes, and they polarize toward the M1 (proinflammatory) or M2 (anti‐inflammatory) phenotype (Gao & Hernandes, 2021; Kwon & Koh, 2020; Moraes et al., 2021). M1 microglia release various proinflammatory factors and oxidative products, such as IL‐1β, IL‐1α, IL‐6, IL‐12, IL‐17, IL‐23, IFN‐γ and TNF‐α. These proinflammatory factors promote chronic neuroinflammation, increase phagocytosis, produce oxidation products, and contribute to neurodegeneration, inhibiting neuronal regeneration and increasing brain damage (Moraes et al., 2021; Orihuela et al., 2016). Oxidation products (e.g., NO and ROS) generated by proinflammatory factors contribute to BBB destruction while promoting the inflammatory response mediated by activated microglia. Moreover, the production of inflammatory cytokines can further activate microglia. Finally, immune cells are recruited from the periphery to the CNS through the release of proinflammatory chemokines (e.g., CCL2, CCL5, CXCL3, CCL12, and CCL13), which amplify inflammatory signals and create a vicious cycle of neuroinflammation (Errede et al., 2022; Prinz et al., 2019; Quaranta et al., 2023).

M2 microglia are neuroprotective and release various anti‐inflammatory factors, such as IL‐4, IL‐10, IL‐13 and TGF‐β, to reduce inflammation, and phagocytose age‐damaged organelles (i.e., cellular debris), misfolded proteins and metabolic fragments, and release neurotrophic factors to promote neuronal healing and neurological function recovery (Cherry et al., 2014; Wolf et al., 2017; Zhang et al., 2017). After brain damage, microglia tend to polarize toward the M1 phenotype, with only a few transient M2 microglia exhibiting disruption of the dynamic equilibrium between proinflammatory and anti‐inflammatory conditions, leading to chronic neuroinflammation and subsequent damage to the brain, which causes brain dysfunction (David & Kroner, 2011).

### 3.2 Neuroinflammation

Neuroinflammation is the immune response of the brain to stimuli such as infection, traumatic brain injury, autoimmunity, or metabolic toxins in the CNS and involves the activation of different types of cells within the CNS, such as astrocytes and microglia (Ebert et al., 2019; Teleanu et al., 2022). During sepsis, neurons can be damaged through a variety of mechanisms; specifically, inflammatory factors and inflammatory signals reach different regions of the brain to induce neuroinflammation through various means, such as the humoral and neural pathways (Castro et al., 2022). Numerous studies have shown that neuroinflammation plays a central role in the pathogenesis of SAE and that an uncontrolled inflammatory response is the main manifestation of sepsis. Neuroinflammation is one of the major causes of brain dysfunction and brain cell death (Schwalm et al., 2014). Various pathological mechanisms in the brain can trigger a neuroinflammatory response, which can ultimately contribute to the dysfunction of various processes.

Hyperactivation of microglia, which are innate immune cells, is a major player in neuroinflammation (Leng & Edison, 2021) and induces a variety of neuropathological disorders, such as SAE, Alzheimer's disease, spinal cord injury, Parkinson's disease and subarachnoid hemorrhage (Jiang et al., 2020; Leng & Edison, 2021; Liu et al., 2022; Shen et al., 2021; Tian et al., 2022). Classically activated (M1) microglia directly or indirectly induce neuropathological changes, such as astrocyte activation, brain endothelial damage, inflammation, synaptic dysfunction, neuronal damage and cell death (Chen et al., 2021; Karunia et al., 2021), through the release of proinflammatory factors, oxidative products, chemokines and complement factors (Yan et al., 2022; Ye et al., 2019), In SAE, microglia activate and subsequently phagocytose neurons, including those in the neuronal cytosol, synapses (Chung et al., 2023; Wu et al., 2023) and myelin sheaths, leading to structural or functional abnormalities in the brain (Jansen et al., 2022; Karunia et al., 2021), which can cause cognitive dysfunction and acute neurological deficits. Therefore, modulation of microglial activation and polarization, which tends to be beneficial, is important for improving the prognosis of a variety of inflammation‐associated neurological disorders, such as SAE.

**FIGURE 1:**
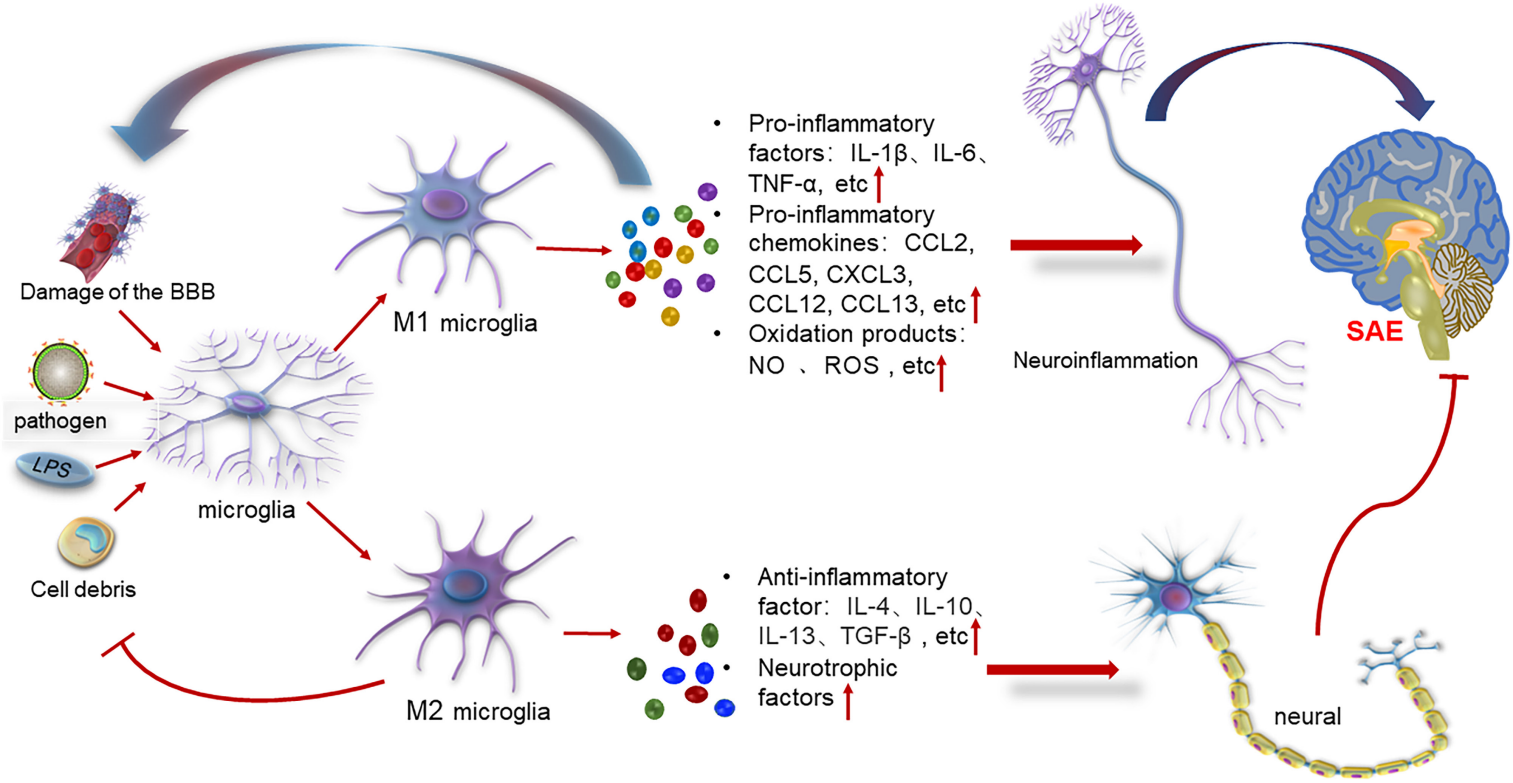
Microglia are polarized toward the M1 and M2 phenotypes by endogenous or exogenous stimuli. M1 microglia releases proinflammatory factors, proinflammatory chemokines, oxidative products, etc., to exacerbate neuroinflammation; inflammatory cytokines and oxidative products in turn further activate microglia and promote blood–brain barrier disruption. M2 microglia exert neuroprotective effects and phagocytose cell debris and misfolded proteins while releasing anti‐inflammatory factors and neurotrophic factors to promote neuronal healing and neurological function recovery.

In summary, the induction of neuroinflammatory responses by the release of inflammatory factors, chemokines, complement factors and oxidative stress products, resulting in damage to the brain parenchyma after the activation and polarization of microglia by endogenous or exogenous stimuli (the transformation of many M0 microglia to M1 microglia), may be a key factor in the development of SAE (Figure 1).

## 4 HOW CAN MICROGLIAL ACTIVATION BE MODULATED TO ALLEVIATE SAE?

### 4.1 Autophagy regulates microglial activation

Autophagy is an important intracellular degradation process (Morishita & Mizushima, 2019) and a highly conserved cellular self‐renewal process in eukaryotes. Cellular autophagy is widespread in eukaryotes and is an important mechanism for maintaining homeostasis and cell survival (Gatica et al., 2018). Many studies have confirmed that autophagy is an important regulator of the inflammatory response (Shadab et al., 2020; Shao et al., 2021, 2022), but the role of autophagy in the inflammatory response in the brain is poorly understood. Several studies have reported that autophagy may be involved in regulating microglial activation or phenotypic transformation, thereby modulating neuroinflammation and neuronal cell death (Hu et al., 2021; Pi et al., 2021). Autophagy plays a key role in regulating microglial activation, and insufficient autophagy may induce microglial hyperactivation and polarization and increase neuroinflammation (Shen et al., 2021).

In neurological disorders, autophagy has been shown to affect microglial phagocytosis by interfering with microglial activation (Li et al., 2021). Inhibition of microglial autophagy promotes the conversion of microglia from the M1 phenotype to the M2 phenotype, counteracts neuroinflammatory responses and thereby reverses brain damage and alleviates cognitive dysfunction (Feng et al., 2022). In another study in which the expression levels of autophagy markers such as microtubule‐associated protein‐light chain 3β (LC3‐II) and autophagy‐associated protein 7 (Atg7) were assessed in microglia, increased microglial autophagy was shown to exert an anti‐neuroinflammatory effect (Lee et al., 2021). Deletion of autophagy‐associated protein 5 (Atg5) in microglia, i.e., insufficient microglial autophagy, promotes microglial activation to induce neurotoxicity and neuroinflammation through activation of the NLRP3 inflammasome via the PDE10A‐cAMP pathway, leading to neurological deficits (Cheng et al., 2020). Thus, increasing microglial autophagy ameliorates cognitive dysfunction and reverses memory deficits.

In recent years, researchers have begun to focus on the effect of autophagy on the polarization of microglia. A study further confirmed that autophagy is inhibited in activated microglia and that this change promotes brain injury‐induced neuroinflammatory responses (Hegdekar et al., 2023). Inhibition of autophagy attenuates microglial phagocytosis, resulting in the accumulation of damaged neurons and an inflammatory response (Beccari et al., 2023). Microglial autophagy may regulate microglial activation through multiple pathways, but these pathways require further exploration.

### 4.2 miRNAs regulate microglial activation

MicroRNAs are highly conserved, single‐stranded noncoding RNAs approximately 22 nucleotides in length that are encoded by endogenous genes; they play a variety of important regulatory roles in cells and are implicated in the development of many pathological diseases (Lei et al., 2022). MicroRNAs (miRNAs) have been found to be aberrantly expressed in a wide range of human diseases, such as neurodegenerative diseases, cancers, diabetes, viral infections, cardiovascular diseases and other diseases. Recently, it was reported that microRNAs are significantly aberrantly expressed in sepsis‐associated organ dysfunction and are thus promising biomarkers for this condition. A review of previous studies revealed at least 122 microRNAs and signaling pathways involved in sepsis‐associated organ dysfunction (Antonakos et al., 2022; Maiese et al., 2022). However, how aberrant microRNA expression regulates various pathological mechanisms in SAE is unclear.

**FIGURE 2:**
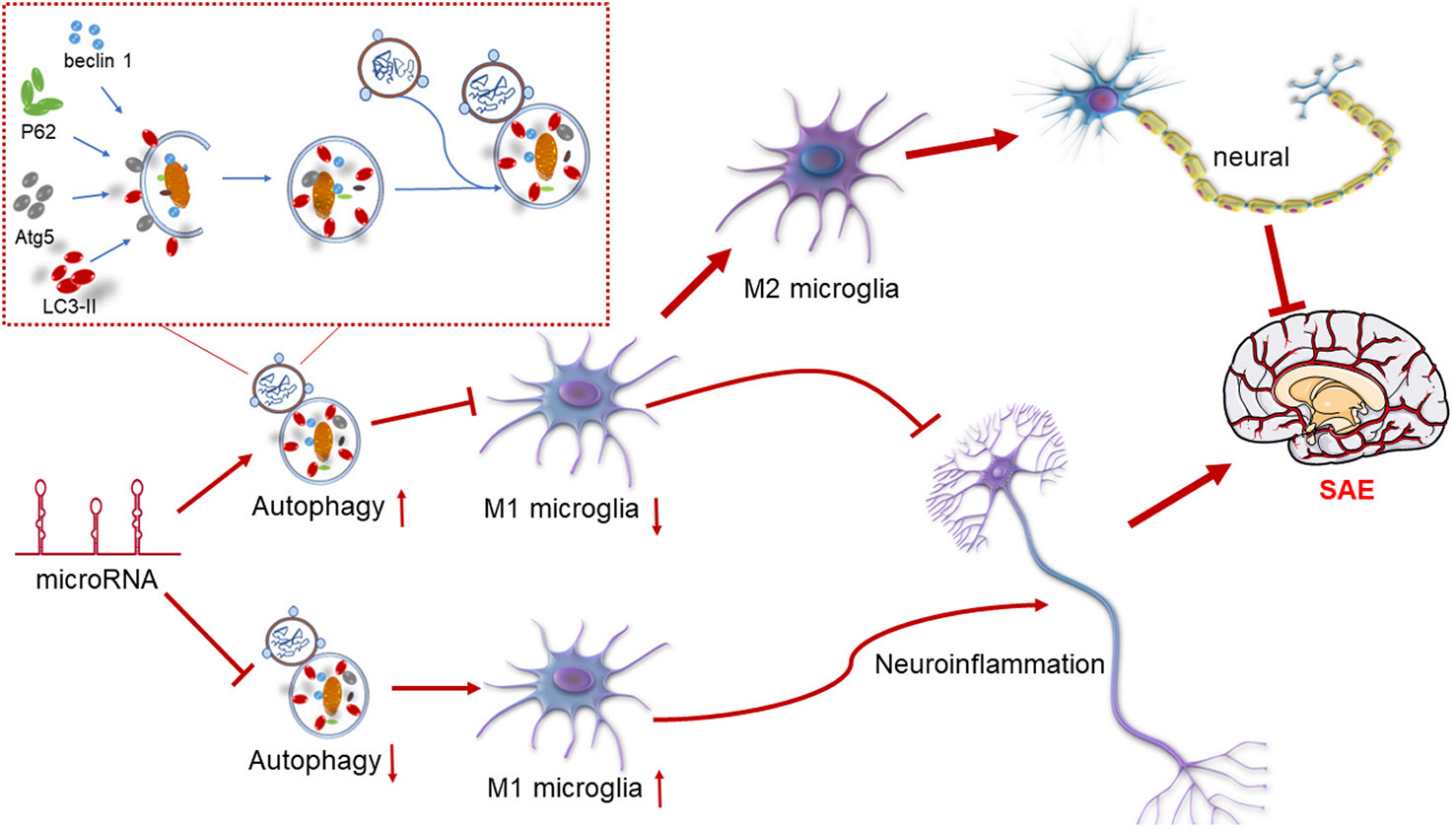
MicroRNA‐mediated regulation of autophagy affects microglial activation, (1) microRNAs promote autophagy, thereby inhibiting microglial activation, and (2) microRNAs inhibit autophagy, thereby promoting microglial activation. Exacerbation of neuroinflammatory responses contributes to the development of SAE. SAE, sepsis‐associated encephalopathy.

Recent studies have confirmed that microRNAs may play a key role in the activation or inhibition of microglia to regulate microglia‐induced inflammatory responses and autophagy. In SAE, microRNAs are among the most important regulators of microglial activation, polarization and autophagy and consequently affect neuroinflammation (Figure 2). microRNA‐375 has been identified as a biomarker of acute inflammation in rats, and microRNA‐375 modulates the JAK2‐STAT3 pathway to regulate the expression of microRNA‐21, which in turn controls the development of sepsis (Sheng et al., 2017; Tang et al., 2021). In a mouse model of Alzheimer's disease, microRNA‐155 was found to be a key regulator of microglial function and microglia‐mediated synaptic homeostasis (Aloi et al., 2023). Downregulation of microRNA‐210 reduces microglial activation and exerts an anti‐neuroinflammatory effect by inhibiting TET2 expression (Li et al., 2023). Moreover, downregulation of microRNA3b‐4p was found to activate microglia in the hippocampus and thus increase neuroinflammation in a mouse model of poststroke depression (Ke et al., 2023). Finally, microRNA‐124 inhibits microglial activation to attenuate the inflammatory response (Chen et al., 2023).

MicroRNAs play an important role in autophagy regulation (Akkoc & Gozuacik, 2020). Overexpression of microRNA‐195 promotes microglial activation by inhibiting autophagy, inducing the release of the proinflammatory cytokines IL‐1β, TNF‐α and iNOS and exacerbating neuroinflammation and neuropathic pain (Shi et al., 2013). It was found that microRNA‐Let7A is involved in the regulation of microglial autophagy. microRNA‐let7A overexpression upregulates the expression of Beclin‐1, LC3‐II and Atg3 in LPS‐treated BV2 microglia (Song et al., 2015). It was reported that in a neonatal rat model of cerebral ischemia and hypoxia, miR‐210 induces the polarization of microglia toward the M1 phenotype partly by targeting SIRT1, which reduces the deacetylation of the NF‐κB subunit p65 and increases NF‐κB signaling (Li et al., 2020). Finally, upregulation of microRNA‐506‐3p was shown to exert neuroprotective and anti‐inflammatory effects, and microRNA‐506‐3p inhibited microglial activation by targeting the CCL2‐CCR2 axis (Jin et al., 2023). In summary, microRNAs can directly or indirectly regulate the polarization of microglia (e.g., microRNAs can regulate microglial activation by regulating autophagy).

Little is known about how microRNAs regulate microglial activation in SAs. Recent studies have reported that downregulation of microRNA‐210 can effectively inhibit activated microglia‐mediated neuroinflammation and significantly alleviate HIE‐induced brain injury (Li et al., 2020). microRNA‐146a‐5p promotes activated microglia‐induced neuroinflammatory responses in the brain by activating TLR7 signaling in a mouse model of multiple microbial sepsis (Zou et al., 2022). MicroRNA‐25‐3p overexpression attenuates the activation of microglia in SAE by regulating the NLRP1/IL‐18β/IL‐4 axis via TLR3 (Luo et al., 2022). The transcription factor YY1 upregulates TREM‐2 expression to promote microglial M2 polarization and alleviate neuroinflammation and behavioral deficits in SAE by inhibiting microRNA‐130a‐3p (Peng et al., 2022). microRNA‐494 can further regulate the activation of microglia in SAE by modulating mitochondrial function (He et al., 2022).

The potential relationship between microRNAs and procalcitonin (PCT) has gradually increased. First, PCT is an acute soluble protein released by the body in response to systemic inflammation, especially bacterial infection, and is an early diagnostic marker of severe bacterial infection and sepsis (Reinhart et al., 2000; Tosoni et al., 2020). PCT, a biomarker of sepsis, has shown good diagnostic accuracy in predicting sepsis in patients with suspected sepsis (Leli et al., 2016). PCT is also used clinically as a marker for assessing the severity of sepsis and plays an important role in guiding antibiotic therapy in SAEs (Carr, 2015; Schuetz & Müeller, 2016). SAE can develop on the basis of severe sepsis, and PCT may also play a role in SAE. MicroRNAs are considered promising biomarkers for SAE and may also serve as therapeutic targets for SAE. Therefore, PCT could also be a therapeutic target for SAE, and a study confirmed that inhibition of microRNA‐497‐3p downregulates PCT expression and exacerbates bacterial pneumonia in mice (Wang et al., 2020). MicroRNA‐125b has been shown to downregulate PCT expression in sepsis patients, ameliorating sepsis (Le et al., 2018; Zhang et al., 2016). In SAE, we investigated whether microRNAs may also ameliorate SAE by modulating PCT expression to reduce microglial activation. Therefore, we hypothesized that microRNAs may be very important therapeutic targets in SAE (Table 1).

**TABLE 1:** MiRNAs may be therapeutic targets in SAE.

miRNA	Role	Reference
miRNA‐210	Downregulation of MicroRNA‐210 reduces microglia activation and acts as an anti‐neuroinflammatory agent by suppressing TET2 expression	Li et al. (2023)
miRNA‐3b‐4b	Downregulation of microRNA3b‐4p activates microglia in the hippocampus to enhance neuroinflammation	Ke et al. (2023)
miRNA‐124	Inhibition of microglia activation by microRNA‐124 attenuates inflammatory response	Chen et al. (2023)
miRNA‐195	MicroRNA‐195 overexpression enhances neuroinflammation by inhibiting autophagy and further promoting microglia activation	Shi et al. (2013)
miRNA‐Let7A	MicroRNA‐let7A overexpression upregulates Beclin‐1, LC3‐II and Atg3 expression levels in LPS‐induced BV2 microglia	Shi et al. (2013)
miRNA‐506‐3P	Upregulated microRNA‐506‐3p has neuroprotective and anti‐inflammatory functions, and it inhibits microglia activation by targeting the CCL2‐CCR2 axis	Li et al. (2020)
miRNA‐210	Downregulation of microRNA‐210 effectively inhibits microglia activation polarization‐mediated neuroinflammation and significantly reduces HIE‐induced brain injury	Jin et al. (2023)
miRNA146a‐5P	microRNA‐146a‐5p acts as an activator of TLR7 signaling to promote microglia activation polarization‐induced neuroinflammatory responses in the brain	Luo et al. (2022)
miRNA‐25‐3P	MicroRNA‐25‐3p overexpression attenuates activated polarization of microglia in SAE by regulating the NLRP1/IL‐18β/IL‐4 axis through TLR3	Luo et al. (2022)
miRNA‐130a‐3P	Inhibition of microRNA‐130a‐3p to upregulate TREM‐2 expression promotes microglia M2 polarization and alleviates neuroinflammatory and behavioral deficits in SAE	Peng et al. (2022)
miRNA‐494	MicroRNA‐494 further modulates activation polarization of microglia in SAE by regulating mitochondrial function	He et al. (2022)

Abbreviation: SAE, sepsis‐associated encephalopathy.

### 4.3 Summary and outlook

SAE is a major threat to the lives of patients with sepsis, especially elderly patients. The pathological mechanisms of SAE are not fully understood, but microglia‐mediated neuroinflammation is known to play a central role in SAE. Despite widespread knowledge about the role of microglia in neurological disorders, many questions regarding the role of microglia in SAE have not been answered. However, further exploration is needed to develop microglia‐targeted treatment strategies for SAE. It is unclear how regulating microglia‐mediated neuroinflammation can counteract cognitive deficits and improve the prognosis in SAE patients. In recent years, autophagy and microRNAs have been found to regulate microglial activation to counteract neuroinflammation, and further studies on the regulation of microglial activation by autophagy and microRNAs will provide new potential therapeutic targets for the treatment of neuroinflammation in SAE.

## AUTHOR CONTRIBUTIONS

NQ, YM, LX, XM and PX performed the literature search, wrote the first draft of the manuscript, and which was critically reviewed by PX. All authors contributed to the article and approved the submitted version.

## FUNDING INFORMATION

This work was supported by the National Natural Science Foundation (Grant Nos.82060359, 82360382) of China; Guizhou Province Social Development Project: Qiankehe [2021] General 088; Key Project of Guizhou Natural Science Foundation: Qiankehe Fundamentals ZK [2022] Key 049; Guizhou Province Excellent Youth Science and Technology Talent Project: Qiankehe Platform Talent [2021] No. 5648; Zunyi Excellent Youth Science and Technology Talent Project: Zunyou Qingke (2020) No. 2; Zunshi Kehe H8 Zi (2020) No. 144; Yuan Ke Zi (2020) No. 13.

## CONFLICT OF INTEREST STATEMENT

The authors declare that the research was conducted in the absence of any commercial or financial relationships that could be construed as a potential conflict of interest.

## ETHICS STATEMENT

Not applicable.
